# Chronic fatigue syndrome: aetiology, diagnosis and treatment

**DOI:** 10.1186/1471-244X-9-S1-S1

**Published:** 2009-10-23

**Authors:** Alfredo Avellaneda Fernández, Álvaro Pérez Martín, Maravillas Izquierdo Martínez, Mar Arruti Bustillo, Francisco Javier Barbado Hernández, Javier de la Cruz Labrado, Rafael Díaz-Delgado Peñas, Eduardo Gutiérrez Rivas, Cecilia Palacín Delgado, Javier Rivera Redondo, José Ramón Ramón Giménez

**Affiliations:** 1 Carlos III Health Institute. Sinesio Delgado, n° 6, 28029, Madrid. Spanish Society of Primary Care Physicians. Narváez, 15 1° Izda, 28009, Madrid, Spain; 2 Spanish Society of Family and Community Medicine. Portaferrissa 8 pral., 08002, Barcelona, Spain; 3 Public Health and Health Management Chair, European University of Madrid. Tajo s/n., Urb. El Bosque, 28670, Villaviciosa de Odón, Madrid, Spain; 4 Coordinating Institution for the National Associations of Fibromyalgia and Chronic Fatigue. Rafael Bonilla 19, local, 28028, Madrid, Spain; 5 Spanish Society of Internal Medicine. Pintor Ribera 3, 28016, Madrid, Spain; 6 Spanish Society of Psychosomatic Medicine and Medical Psychology. Avda. de los Angeles, 14 Portal 2 - 2° C, 28223, Pozuelo de Alarcón, Madrid, Spain; 7 Spanish Association of Paediatrics. Aguirre 1, bajo derecha, 28009, Madrid, Spain; 8 Spanish Society of Neurology. Via Laietana, 23, entlo A-D, 08003, Barcelona, Spain; 9 Spanish Society of Physiotherapy. Calle Rodriguez Marín 69, bajo D, 28016, Madrid, Spain; 10 Spanish Society of Rheumatology. Marqués de Duero,5, 1°, Madrid, 28001, Spain; 11 Carlos III Health Institute. Sinesio Delgado, n° 6, 28029, Madrid, Spain

## Abstract

Chronic fatigue syndrome is characterised by intense fatigue, with duration of over six months and associated to other related symptoms. The latter include asthenia and easily induced tiredness that is not recovered after a night's sleep. The fatigue becomes so severe that it forces a 50% reduction in daily activities. Given its unknown aetiology, different hypotheses have been considered to explain the origin of the condition (from immunological disorders to the presence of post-traumatic oxidative stress), although there are no conclusive diagnostic tests. Diagnosis is established through the exclusion of other diseases causing fatigue. This syndrome is rare in childhood and adolescence, although the fatigue symptom *per se *is quite common in paediatric patients. Currently, no curative treatment exists for patients with chronic fatigue syndrome. The therapeutic approach to this syndrome requires a combination of different therapeutic modalities. The specific characteristics of the symptomatology of patients with chronic fatigue require a rapid adaptation of the educational, healthcare and social systems to prevent the problems derived from current systems. Such patients require multidisciplinary management due to the multiple and different issues affecting them. This document was realized by one of the Interdisciplinary Work Groups from the Institute for Rare Diseases, and its aim is to point out the main social and care needs for people affected with Chronic Fatigue Syndrome. For this, it includes not only the view of representatives for different scientific societies, but also the patient associations view, because they know the true history of their social and sanitary needs. In an interdisciplinary approach, this work also reviews the principal scientific, medical, socio-sanitary and psychological aspects of Chronic Fatigue Syndrome.

## Background

The chronic fatigue syndrome (CFS) is fundamentally characterized by intense fatigue of unknown cause, which is permanent and limits the patient's functional capacity, producing various degrees of disability.

In medical terminology, fatigue is the early onset of tiredness after an activity has been started; it is a sensation of exhaustion or difficulty to carry out physical or intellectual activities, without recovery after a period of rest. Fatigue has been categorized as recent fatigue, prolonged fatigue and chronic fatigue, according to the time of evolution (less than one month, more than one month and more than six months, respectively) [1].

It is advisable to differentiate fatigue from other medical concepts with which the symptom is often confused: first, from asthenia, defined as the lack of strength or feeling of inability to carry out daily tasks, which is more intense at the end of the day, and usually improves after a period of sleep; second, from weakness, which is the reduction or loss of muscular strength, and the key symptom in muscular diseases.

In addition to fatigue, CFS is associated to a wide spectrum of symptoms, including arthralgias, muscle pain, headaches, anxiety, depressive symptoms, cognitive disorders, sleep disorders, or intolerance to physical exertion, among the most frequent [2,3].

The little understanding of CFS aetiopathogeny, together with the difficulties to achieve an objective and quantitative assessment of the symptoms that affected patients have, has prevented for a long time the establishment of a diagnosis [4]. A consequence of such a problem is the variety of names CFS is known for, including allergic encephalomyelitis, immune dysfunction syndrome, neuroendocrine immune dysfunction syndrome, post viral syndrome, Iceland disease, neurasthenia, and Royal Free disease, among others [5].

The various criteria established in recent years have allowed a more accurate delineation of CFS, and this has contributed to a better understanding of its clinical picture, and potential therapeutic interventions [1].

CFS is, therefore, a complex, chronic disorder of unknown aetiology, characterized by the presence of intense and disabling fatigue (physical and mental), with a clinical course and without any apparent cause, which interferes with daily activities, does not decrease with rest, worsens with exercise, and is usually associated to systemic, physical and neuropsychological manifestations [6,7].

The aetiology, diagnosis and therapeutic options for chronic fatigue syndrome in adults and pediatric patients are discussed below.

### The aetiology and the pathogenic mechanisms of CFS

As the criteria for CFS diagnosis are not based on the understanding of aetiopathogenic mechanisms, some patients present similar clinical manifestations but are diagnosed with other conditions because fatigue is not the primary symptom. Some of those conditions are fibromyalgia, irritable bowel syndrome, and temporomandibular joint syndrome. Furthermore, in addition to sharing several symptoms with CFS, currently available evidence suggests that those diseases also share similar pathophysiologic mechanisms [8,9].

Although the aetiology and the pathogenic mechanisms of CFS are not fully understood, several hypotheses have been postulated and described below, being the disorders of the central nervous system neuromodulator the one supported by more evidence to explain the possible pathogenic mechanisms involved in CFS [5].

#### Infectious theory

Epstein Barr virus, *Candida albicans, Borrelia burgdorferi*, Enterovirus, Citomegalovirus, Human Herpesvirus, Espumavirus, Retrovirus, Borna virus, Coxsackie B virus, and hepatitis C virus (HCV) have been associated to CFS, but their pathogenic relationship with the syndrome has not been demonstrated [10].

#### Immunological theory

Although different disorders have been found in the immune system or its function, currently there is no scientific evidence to attribute the cause of this syndrome to a primary disorder of the immune system. There are a large number of studies on immune disorders in the CFS assessing identical parameters, but they frequently yield contradictory results [11-14].

#### Neuroendocrinological theory

Several disorders in the hypothalamic-pituitary-adrenal axis (HPA) and in the production of related hormones have been found in CFS, as well as a disorder of the regulating mechanisms of the autonomic nervous system. It is currently known that the relationships between the different parts of the nervous system are mediated by neurotransmitters and that their disorders lead to unbalanced functioning of certain structures and to the development of well known diseases. Many of the clinical features in patients with CFS are similar to those found in patients with fibromyalgia, and it can therefore be postulated that the physiopathological mechanisms are probably similar in both conditions.

In patients with fibromyalgia, the research on neurotransmitter disorders has started to yield positive findings, and it is known that different clinical manifestations will appear according the type and the site of action of affected neurotransmitters [8,15].

### Prevalence and clinical features

It is difficult to establish the prevalence of CFS, since it depends on the diagnostic criteria used and the study population. Initial research suggested a prevalence between 0.002% and 0.04%. [16,17]. However, latest epidemiological studies in the USA and in the United Kingdom show prevalence rates ranging from 0.007% to 2.5% of the general population. [18] These rates increase up to 0.5-2.5% when the population assessed includes individuals seen in primary care facilities instead of the global population. [19] In the United Kingdom, according the Oxford criteria [20], the prevalence in the global population has been estimated in 0.6%. In Japan the prevalence has been found to be 1.5% in the general population[21,22]. Thus, the prevalence in the general population appears to be much higher than previously indicated. Even with strict criteria for CFS, it is estimated that approximately 1% of the adult population experiences this condition. Interestingly, a large part of this group remains unrecognized by the general practitioner. A striking similarity in lifestyle pattern between SF, CF and CFS calls for further research. [23]

CFS mainly affects young adults from 20 to 40 years, although the symptoms also exist in childhood, adolescence and in the elderly [10]. It has a 2-3 times higher prevalence in women than in men. No evidence exists showing that any socio-economic group is more affected than others [5].

The typical CFS case occurs acutely, and even suddenly, usually in a previously healthy person. Initially, fever, sore throat, cough, muscular pain and fatigue are the typically predominant symptoms; digestive symptoms such as diarrhoea are less common. This initial process resolves with intense tiredness as a sequel. The cardinal or key symptom is fatigue, essential for diagnosing the condition. Fatigue in CFS is characterised by not being secondary to excessive activity, with no improvement associated with rest and worsening with stress, and directly resulting in persistent disability (physical and mental) [7].

The chronic symptoms develop later [24], persisting for weeks or months. Predominant symptoms vary for the individual patient, and include fatigue, fever or intermittent dysthermia, migratory arthralgias, generalised musclar pain, pharyngitis or sore throat, headache, tender cervical or axillary lymph nodes, and other less common symptoms.

Fatigue is usually associated to neurocognitive and sleep disorders. Patients have difficulty in concentrating, insomnia or hypersomnia, and occasionally depression. Palpitations, thoracic pain, night sweating, or weight loss/increase are less common [10].

In general, clinical evolution is characterized by regular and even seasonal recurrences. Each outbreak can be different from the previous one, and periods between each recurrence are rarely completely asymptomatic [1]. CFS's symptomatology worsens with physical or emotional stress, interfering or limiting previous activities (including family, work, and social activities); in some cases, patients may need help for their basic daily activities.

The main co-morbidity is related with psychiatric disorders, such as depression or anxiety, with an approximate incidence of 28% in the Western population [25,26].

### Diagnosis

As there is no pathognomonic sign or specific test for CFS, the diagnosis of the syndrome is clinical. Other causes of fatigue should be ruled out, through a complete and detailed medical history, focused on the characteristics of fatigue, delineating its form and time of onset, duration, triggering factors, relationship with rest and physical activity, and the degree of limitation of the patient's regular activities. Furthermore, targeted interrogation will collect the symptoms in the osteomuscular, neurovegetative and neuropsychological domains. Thus, chronic fatigue should be differentiated from debilitation, exercise intolerance, sleepiness, or loss of motivation and stamina.

The presence of psychiatric disorders (anxiety, depression) should be included in the personal history as well as possible non-infectious precipitating factors (organophosphorous insecticides, solvents, CO, multiple chemical hypersensitivity, sick building syndrome, situations that disturb sleep, etc.), and prior history of allergies. This information should be included to rule out other alternative diagnoses such as infections, neoplasias, depression or sleep disorder.

Specific exploration is required for the musculoskeletal system (strength, reflexes and muscular tone), the neurological system (looking for any neurological deficit), the cardiovascular and respiratory systems (anaemia and cardiac insufficiency), the endocrinological system (thyroid gland disorders), the immune system (tender cervical, axillary or inguinal lymph nodes) and the gastrointestinal system. Physical findings are usually unspecific, and a large variety of signs can be found, such as pharyngeal soreness, fever, tender posterior cervical or axillary lymph nodes, muscular tenderness on palpation, and, occasionally, rash.

Currently, there are no specific biological or morphological markers to establish *per se *the diagnosis of the CFS, and therefore none of the alterations that can be found are useful for diagnosis. Diagnostic criteria basically arise as a research requirement, but their limitations for actual clinical practice must be accepted.

The Centres for Disease Control and the CFS International Study Group proposed in 1994 an international diagnostic criteria (Table 1) [27]. Their main objectives were to increase the sensitivity of the previous classification, and to offer a more accurate definition of the condition, in order to achieve a more consistent clinical diagnosis and use it as a research tool. The international criteria are based on the fulfilment of two major criteria (chronic fatigue causing incapacity, lasting more than 6 months, and the exclusion of associated medical and psychiatric conditions), as well as the concurrence of a series of criteria, reducing the symptoms from 11 to 8: these criteria are based on symptoms, particularly rheumatological and neuropsychological symptomatology.

**Table 1:** Diagnostic criteria for chronic fatigue syndrome

1.-Persistent chronic fatigue (at least 6 months) or intermittent, unexplained chronic fatigue, which relapses, or with a definite start, and is not the result of recent exertions. Does not improve with rest. Results in a significant reduction in the patient's previous normal activity.	
2.-Exclusion of other diseases that may cause chronic fatigue. Four of the following minor criteria (signs or symptoms) must be present concurrently for six months or longer, after the onset of fatigue:	

Minor criteria (Signs and symptoms)	1-Recently impaired memory or concentration.
	
	2.-Odynophagia
	
	3.-Painful axilar or cervical adenophatias
	
	4.-Myalgias
	
	5.-polyartralgias without phlogosis
	
	6.-Headache with a new pattern or seriousness.
	
	7.-sleep which does not improve by resting.
	
	8.-Discomfort post effort > 24 hs.

#### Diagnostic protocol for patients with suspected CFS

**Figure 1:**
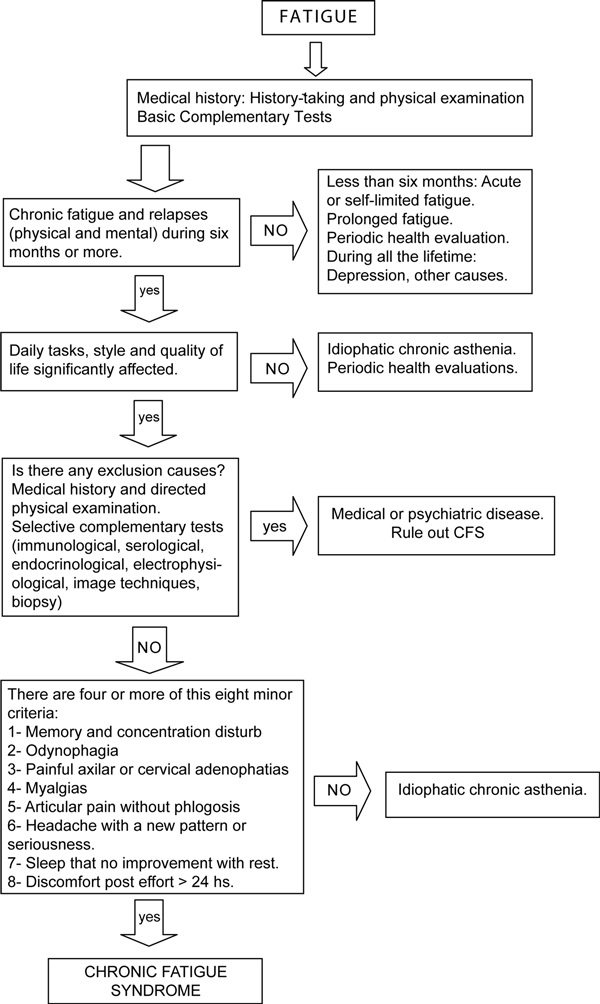
**Diagnostic protocol for patients with suspected CFS**.

Figure 1 details the algorithm for CFS diagnosis [28]. Conditions that exclude the diagnosis of CFS are: psychiatric disorders, such as major depression, schizophrenia, eating disorders (anorexia, bulimia), bipolar disorder, alcohol or other substance abuse, in addition to morbid obesity, and active medical diseases, either non-treated or without a completely established resolution.

#### Prognosis

There is an average time of 5 years from the beginning of the symptoms to the diagnosis of the syndrome, with total recovery rates between 0% and 37%, and improvement between 6% and 63% [29]. Younger patients and those without concomitant psychiatric diseases show the best prognosis, although other studies have estimated that the rates for both groups are similar [30].

### Assessment methods

There is no single tool for the assessment of patients with CFS that allows a global appraisal of the clinical manifestations and the impact of the disease on patients. There are specific questionnaires, according the feature to be measured, that can provide useful information on specific issues. A summary of them can be found in the systematic review by Bagnall et al [31]. However, the most useful way of collecting information is with interviews and patient diaries. Interview can include patient self-records, questionnaires and scales for functional assessment, such as "Karnofsky Performance Scale, Medical Outcomes Study Short-Form General Health Survey" (SF-36^®^) [32] and Sickness Impact Profile (SIP) [33]. Interviews should be repeated periodically in time.

Self-records and scales are excellent references and help the therapist to assess the patient's daily activities, general functioning and the degree of disability.

The scale is useful for the patient because they can fill in a hierarchy/severity scale their symptoms during the initial visit, and then, approximately every 6 months. This scale categorizes both the severity of the symptom and the aggravating factors.

Daily activity/functional capacity scale is also a useful tool. In this case the patient is asked to make a diary of all their daily activities and periods of rest for a week.

#### Symptoms assessment

The easiest way to measure *pain *in the locomotive apparatus is with an Analogical Visual Scale (AVS), especially when trying to assess the pain that a patient has experienced during a given period of time [34].

To assess fatigue, one of the most used tools is the Multidimensional Fatigue Inventory [35], a 20-item questionnaire that measures global, physical and mental fatigue, and decrease in activity and motivation.

When the patients have difficulties for carrying out physical exercise it is important to quantify the degree of impairment. The most objective methods are based on determining the aerobic capacity of patients, usually with spiro-ergometric tests, expired gases/heart rate are measured, and work load quantified. Other alternative methods that offer semi-quantitative measures, and are often used, are the 6-minute running test [36], measuring the strength of certain muscular groups, and the degree of mobility of the column or the peripheral joints.

The assessment of disability is complex due to the fact that the clinical diagnostic criteria have not been validated in the medical-legal framework, to the lack of any objective proof of existence as well the lack or low medical-legal performance of validated instruments to quantify the disability associated with CFS. There is a big barrier for fulfilling two major conditions in the assessment of the disability, namely objective evidence of the impairment and the absence of data corroborating the severity of the pain [37].

## Treatment

The therapeutic approach to CFS is complex and requires a combination of different therapeutic modalities. In recent decades, many therapies for CFS have been examined, but the only one that has demonstrated a significant efficacy in patients diagnosed with CFS, together with gradual physical exercise, is cognitive behaviour therapy, which has been intensely developed in recent years [38-40]. A 2008 Cochrane review found that 40% of patients reported improvements in fatigue after cognitive behavioural therapy compared with 26% in usual care at the end of treatment. At follow-up, 1-7 months after treatment ended, when people who had dropped out were included, there was no significant difference between the two groups. [41]

Cognitive therapy as a therapeutic modality for CFS comprises a series of techniques, based on the principles of behaviour modification and the cognitive theory, aimed at strengthening the modification of thoughts and behaviour related to the patient's symptoms and distress [42]. Most protocols developed for this treatment modality are based on three key factors: programmed physical exercise, control and coping with disease-associated stress, and cognitive restructuring [43].

Regarding to prescription of appropriate exercise schedules, there is no consensus for patients with CFS. However, it has been demonstrated that gradual exercise programmes are beneficial for some patients, improving both their physical work and the psychological and cognitive aspects. The main objective of the exercise programme is the progressive prevention of physical deterioration and optimizing the functional capacity, looking for an improvement of the patient's quality of life [44].

Many pharmacological therapies have been used for treating CFS. However, there are very few publications on randomised clinical trials with drugs, and the quality of the available studies is not good [45-47]. On the other hand, since the course of CFS is highly fluctuating, with alternating periods of improvement and deterioration, it has been recommended that any therapeutic modality should comply with several requirements to consider the study as methodologically adequate.

*Ampligen*, an antiviral agent, has been used recently in the treatment of this disease [48-50]. This agent is a stimulator of interferon production, which reduces the levels of RNasaL. Available results from clinical trials show modest improvements, but results need to be verified. The FDA currently considers Ampligen an experimental therapy, and has not approved it for general use [51], although an open clinical trial is being carried out with this drug.

## Chronic fatigue syndrome in paediatric patients

It can be stated that CFS is rare in childhood and adolescence: only 0.06% to 0.32% of children from 5 to 15 years of age fulfil the US Centers for Disease Control and Prevention (CDC) criteria for CFS [51].

Although the CDC criteria is mostly followed [27], different research groups accept that, although fatigue for at least 6 months is required to establish the diagnosis, shorter periods of debilitating fatigue should be considered in adolescents or school-aged children [52]. Since the adjective *debilitating *is the disease's main feature, it is highly likely that shorter periods of 3 months, or even 4-6 weeks, should be considered when associated with absenteeism. This factor is so important that the primary care paediatrician or general practitioner (GP) should suspect CFS whenever it is present, although the clinical picture does not fulfil more stringent criteria.

The Australian Clinical Guide, sponsored by the Royal Australasian Collage of Physicians, includes issues such as the definition of fatigue, its assessment, particular characteristics when the condition affects children and adolescents, and its associated symptoms. But the guide goes beyond major and minor inclusion or exclusion criteria [5]. In contrast with the CDC criteria, this guide emphasizes as a key issue the patient feeling symptomatically ill after a minimum physical or mental effort. In addition, it offers clear explanations for neurological and neuroendocrine disorders, as well as the autonomic and immune manifestations of CFS.

The obsessive tendency to rigidly classify children and adolescents with highly stringent criteria is inappropriate, even though such criteria are internationally accepted. Table 2 summarizes the differences noted in CFS between adults and children.

**Table 2:** Adult/Children CFS differences

**Age**	**Fatigue**	**Symptoms**	**Psychiatric profile**	**Prevalence** **%**	**Sex** **M/F**	**Triggering factor**
Adult 20-40	>6 months	Odynophagia Painful adenophatias Myalgias cephalea	Post-anxiety disorders Depression	0.006-2.5	2-3/1	Flu Cold Serious disease

Child 5-15	>3-6 months	Episodic tension cephalea Recurrent abdominal pain Tachycardia Orthostatic hypotension	Sadness Hyperactivity (initial phase) Fatigue	0.06-0.32	2.5/1	Traumatism Sport failure Mild disease

Children and adolescent patients, although they do not strictly fulfil the CDC criteria, will benefit from an early intervention, with a substantially more adequate therapeutic response [53]. This fact urges primary care paediatricians and GPs to make a presumptive CFS diagnosis resulting in early intervention. Nevertheless, Davies et al assessed the clinical presentation of CFS in children younger than 12-year-old, based on the Royal College of Paediatrics and Child Health criteria (less stricter criteria compared to CDC adult criteria) and found that these children were very disabled, with mean school attendance of just over 40%. But, when compared to adolescent patients, clinical assessment was very similar. In addition, younger patients (24/26) also fullfilled the CDC criteria [54].

In epidemiological studies including children and adolescents, prevalence of 8.7 cases per 100,000 with fatigue, and 2.7 per 100,000 people with CFS are found according to a study published by Jordan et al [55]. It must be assumed that, since there are no accurate and realistic criteria for the diagnosis of CFS in adolescents and children, there are no accurate epidemiological data either.

When analysing the psychological aspects in adolescents who fulfil the CFS diagnostic criteria, more than 1/3 have psychiatric diagnoses at the same time, particularly depression, and less often, generalized anxiety disorder [56]. A prospective, community-based study in the UK found an incidence of 0.5%for CFS in 11 to 15-year-old adolescents, using the CDC criteria, and identified anxiety/depression, conduct disorder, older age and female gender as risk factors for the development of CFS [57]. Several studies show that adolescents with CFS internalize somatic symptoms more intensely and are more disabled than other adolescents diagnosed with chronic diseases with bad prognosis, such as cancer, cystic fibrosis, or juvenile idiopathic arthritis [58].

### Prognosis

Twenty percent of patients in Bell's study [53] continue to consider themselves ill with limitations or disability even 13 years after the onset of the syndrome; although 8% of children find their outcome satisfactory. Although is difficult to differentiate children with CFS from those who are just chronically tired according to the duration of symptoms, in this study there is no correlation between the degree of recovery and age of diagnosis, sex, or clinical features at presentation. The educational impact of the disease is closely related to the outcome; 23% of patients miss school from 1 to 6 months, 8.6% from 6 to 12 months, 5.7% from 1 to 2 years, and 8% do not recover at all after 13 years of follow-up. A follow-up study involving 28 patients, aged 7 to 17 years, highlights the need for early recognition and diagnosis of chronic fatigue syndrome in young people and the importance of continuing paediatric support to reduce symptom persistence in the sensitive recovery period. Maintaining school attendance by close liaison between health and education services both before and after diagnosis and treatment is also vital if long-term morbidity is to be reduced. It should be noted that 15 patients experienced difficulties when returning to school [59]. In another study including 42 children diagnosed with CFS with 1 to 4 years of follow-up after the initial medical intervention, 43% considered themselves cured, 52% improved and 5% showed no changes in their condition [60].

The best predictor for good outcome is still the amount of school time lost during the first 4 years of the disease. Teachers, primary care doctors, paediatricians, psychotherapists and social workers, should be aware of this to achieve the best results and meet the best life expectations for children suffering from this syndrome.

#### Treatment

In adolescents, cognitive behaviour therapy combined with other group therapies that promote treatment compliance and sharing experiences or thoughts with other adolescents is very useful, even when they do not have identical pathologies. It must be noted that there is very limited evidence on cognitive therapy in adolescents. Some non-controlled studies suggest that this therapy reduces fatigue in young people [58,61] with early intervention.

When assessing therapies, it must be remembered that exercise temporarily worsens the symptoms and the times for rest, naps, leisure time, or outdoors activities should be established jointly with the adolescent.

In this chronic situation, reassurance for both adolescents and parents is the key factor that will determine the success of the therapeutic interventions, strengthening the self-confidence and increasing the adherence to the prescribed therapies started, and avoiding the organic versus psychiatric debate.

## Chronic fatigue syndrome in healthcare

### Healthcare cost associated to CFS

It is difficult to estimate the costs imposed by CFS on healthcare. There are few studies evaluating the use of healthcare resources by these patients [62,63].

Although many people suffering CFS continue to work despite of their illness for economic reasons and social prestige, this represents an annual global loss of productivity of approximately €6,900 million, or what is the same €15,200 per patient and year. These figures are comparable to the losses caused by other diseases, such as digestive system-related conditions or infectious and parasitic diseases [1,64], suggesting that the CFS can be included with other chronic processes among the highest healthcare and socioeconomic burdens.

### Healthcare management

Management of CFS should start with a correct and adequate diagnosis and patient care; primary care healthcare professionals should obtain a careful medical history and a complete physical examination. This context gives the best thruway to the healthcare system due to its easy access, and its knowledge and close relationship with people.

Primary care staff should be appropriately trained, and capable of explaining the problem, as well as the available therapeutic options to the patients [5].

After the initial diagnostic suspicion, and although the burden of patients' follow-up can be perfectly accomplished in the primary care context, it is recommended that patients are referred to a second level of specialised care for confirmation of the diagnosis and treatment guidance. Since in CFS medical specialities are involved in care and treatment (rheumatology, internal medicine, psychiatry, etc.), such specialists should also receive adequate training. Cooperation and coordination between primary and specialised care is basic for the correct management of CFS. Occasionally, the intervention of the physiotherapist or psychologist in the treatment is also necessary, and adequate training should also be offered to these healthcare professionals.

In addition to a timely and appropriate diagnosis, patients with CFS usually require individualized management programs, as well as long-term follow-up. Although healthcare professionals are mostly responsible for the latter, the collaboration of the patient's relatives and friends is also essential. It is therefore necessary to train them, with the objective of reducing the patient's anxiety and strengthening the very valuable therapeutic alliance [5]. All this can considerably improve the prognosis of the condition. The particular social context of each patient and the functional repercussion should also be recognized and assessed [65].

Finally, as CFS is not well known, the contribution of enough funds for research is also necessary, and the regulated identification and management of patients or the creation of adequate records by the healthcare system would be a very useful intervention.

### Legal aspects

Giving advice to a person with CFS in medical-legal matters can be very complex and should be done by a qualified, experienced specialist. The notion of "permanent" disability is problematic, as many people with CFS gradually improve. In patients seriously disabled, who have been unable to work for more than five years, the probability of significant improvement in 10 years is less than 10-20%. This can be considered "permanent disability" in medical-legal terms [5].

As explained above, CFS is a highly disabling condition in some patients, frequently requiring legal support for managing possible social aids, handicaps or even disabilities. Ancillary personnel or social workers in healthcare centres and city councils should provide information and advice to the patients when they need it. It will also be necessary for the administrators to adapt the help to each case and the training of their personnel to become familiar with the CFS, avoiding excessive litigation when looking for social aids.

### Educational aspects

A good interdisciplinary team for the diagnosis and treatment of CFS, together with personalised care will enhance the patient's improvement. The person suffering the disease wants to know what can be done to improve and adapt to the upcoming changes.

Patient education should always be supported by the healthcare sector, and may be grouped in two different categories: therapeutic and related to the patient's social setting.

## Conclusion

CFS is a chronic process that becomes a social disease due to the incapacity that it causes in the person who suffers to continue to fulfill their work, social and family responsibilities.

The specific characteristics of the symptomatology of patients with CFS require a rapid adaptation of the educational, healthcare and social systems to prevent the problems derived from current systems. The lack of adequate care for these issues is causing serious difficulties, shortages and even rejections in areas as essential as education, social integration and coexistence, work insertion, and integrated care and medical management.

At present, no curative treatment exists for patients with CFS. Treatment objectives must be focused on improving the clinical manifestations, maintaining the functional capacity and quality of life, and developing a tailored programme, providing each patient with the maximum perception of improvement. Patients with CFS require multidisciplinary management due to the multiple and different issues affecting them. This multidisciplinary management requires coordination between the different specialists, which leads to the need for the existence of an Action Protocol to establish the intervention procedure according to the needs of each patient.

As mentioned above, CFS is disabling in some patients. In these cases, all the support measures recognised in current legislation should be applied. The most important issues to overcome are the difficulties of access to employment, timetable flexibility and ergonomic assessments in order to adapt the work post.

## Competing interests

The authors declare that they have no competing interests.
